# Identifying a Neuromedin U Receptor 2 Splice Variant and Determining Its Roles in the Regulation of Signaling and Tumorigenesis *In Vitro*

**DOI:** 10.1371/journal.pone.0136836

**Published:** 2015-08-28

**Authors:** Ting-Yu Lin, Wei-Lin Huang, Wei-Yu Lee, Ching-Wei Luo

**Affiliations:** Department of Life Sciences and Institute of Genome Sciences, National Yang-Ming University, Taipei, Taiwan; University of Miami, UNITED STATES

## Abstract

Neuromedin U (NMU) activates two G protein-coupled receptors, NMUR1 and NMUR2; this signaling not only controls many physiological responses but also promotes tumorigenesis in diverse tissues. We recently identified a novel truncated NMUR2 derived by alternative splicing, namely NMUR2S, from human ovarian cancer cDNA. Sequence analysis, cell surface ELISA and immunocytochemical staining using 293T cells indicated that NMUR2S can be expressed well on the cell surface as a six-transmembrane protein. Receptor pull-down and fluorescent resonance energy transfer assays demonstrated that NMUR1, NMUR2 and this newly discovered NMUR2S can not only form homomeric complexes but also heteromeric complexes with each other. Although not activated by NMU itself, functional assay in combination with receptor quantification and radio-ligand binding in 293T cells indicated that NMUR2S does not alter the translocation and stability of NMUR1 or NMUR2, but rather effectively dampens their signaling by blocking their NMU binding capability through receptor heterodimerization. We further demonstrated that NMU signaling is significantly up-regulated in human ovarian cancers, whereas expression of NMUR2S can block endogenous NMU signaling and further lead to suppression of proliferation in SKOV-3 ovarian cancer cells. In contrast, in monocytic THP-1 cells that express comparable levels of *NMUR1* and *NMUR2S*, depletion of *NMUR2S* restored both the signaling and effect of NMU. Thus, these results not only reveal the presence of previously uncharacterized heteromeric relationships among NMU receptors but also provide NMUR2S as a potential therapeutic target for the future treatment of NMU signaling-mediated cancers.

## Introduction

G protein-coupled receptors (GPCRs) constitute the largest family of membrane receptors in vertebrates and have more than 800 members found in the human genome [1]. Unlike other receptors for growth factors or cytokines that contain only a single transmembrane domain, the heptahelical transmembrane structure of GPCRs allows complicated rearrangements and is thought to be adequate for signal conduction across the cell membrane [2]. Therefore, GPCRs were initially perceived as monomeric entities. However, growing evidences accumulated over the past two decades indicate that they can form homomeric or heteromeric complexes in intact cells [3–5].

It has now been widely accepted that many GPCRs are able to form homomeric complexes, in which ligand affinity and specificity will be altered due to the conformational change induced by intermolecular interactions within the receptors [3, 6]. In contrast, the functions of GPCR heterodimerization are more enigmatic and seem to vary with the receptor types. It has been demonstrated that GPCR heterodimerization is involved in receptor stability, such as controlling trafficking efficiency and internalization rate. For example, heterodimerization between metabotropic γ-aminobutyric acid B receptors 1 and 2 will mask the endoplasmic reticulum (ER) retention signal of metabotropic γ-aminobutyric acid B receptor 1 and thus facilitates the correct transportation of this functional receptor complex to the plasma membrane [7]. On the other hand, β1 adrenergic receptor is able to stabilize the β2 adrenergic receptor on the plasma membrane through heterodimerization and as a result there is inhibition of the agonist-promoted internalization of β2 adrenergic receptor [8]. In addition to these interactions, the heterodimerization process has also been proposed to affect ligand selectivity and downstream G protein coupling of GPCRs. For example, the heterodimeric complex formed by CCR2 and CCR5 exhibits synergistic agonist binding and is able to trigger a calcium response that is much more sensitive than either receptor alone [9]. Not only conducting both receptor-associated signaling pathways, the forming CCR2/CCR5 heterodimer also recruits dissimilar signaling complexes such as G_q/11_. Likewise, similar effects have also been reported for the heterodimeric κ/δ opioid receptors, where the heterodimer's ligand-binding and functional properties are quite distinct from those of either receptor alone [10]. Of particular interest is the fact that not all GPCRs are able to form heteromeric complexes when they are co-expressed together. Although no rule can accurately predict as yet what GPCRs are able to form heterodimers, it seems that GPCRs have a high preference to heterodimerize with evolutionarily close members [11, 12].

Neuromedin U (NMU) is a highly conserved neuropeptide in mammals and is able to mediate its signaling through two different GPCRs, designated NMUR1 and NMUR2 [13]. These two receptors have different tissue distributions and, as a result, NMU can exert distinct physiological effects on diverse organs locally. NMUR1 is expressed in diverse peripheral organs with relatively high levels in the small intestines and lung [14], where NMU signaling has a role in stimulating smooth muscle contraction and controlling blood pressure [15]. In contrast, NMUR2 is predominantly expressed in the central nervous system, such as the hypothalamus, and also the female ovary and uterus [16]; in this case NMU signaling can play important roles in controlling energy expenditure and reproductive development [13, 17, 18]. Nevertheless, both receptors have also been found to be co-expressed in many organs [19–21], where their functions have not yet been well characterized. Intriguingly, the conservation of certain exon-intron boundaries and the relatively high sequence homology between NMUR1 and NMUR2 suggest that these two receptors are evolutionarily related and may have arisen from a gene duplicative event in the past [22]. This raises a puzzle that whether they can heterodimerize with each other to modulate NMU signaling synergistically.

Surprisingly, during PCR amplification of *NMUR2* from the human ovarian cancer cDNA, we identified a dominant splice variant that lacked the third exon, which we have named *NMUR2S*. Consequently, the encoded sequence is predicted to give rise to a six-transmembrane protein. We have therefore explored NMUR2S’s expression characteristics, its dimerization relationship with NMUR1 and NMUR2, and its effects on NMU signaling in more details.

## Materials and Methods

### Ethical statements and clinical materials

The 10 paired ovarian cancers and matched adjacent normal tissues were collected from patients who underwent surgery at Chang Gung Memorial Hospital Linkou Medical Center. Written informed consent was signed and obtained from all participants. The study protocol was approved by the research ethics committee of Chang Gung Memorial Hospital Linkou Medical Center, Chang Gung University (approval number 97-0675C). All the clinical materials were frozen and stored at -80°C for further real-time quantitative PCR and immunohistochemical analysis.

### Reagents and peptides

DMEM medium, RPMI 1640 medium, penicillin, streptomycin, glutamine and zeocin were purchased from Invitrogen (Carlsbad, CA). Human NMU peptide was obtained from Phoenix Pharmaceuticals (Burlingame, CA). The iodination kit was from Pierce Thermo Scientific (Rockford, IL). Rabbit anti-phospho-ERK1/2 antibody and rabbit anti-ERK2 antibody were purchased from Cell Signaling Technology (Danvers, MA) and Santa Cruz Biotechnology (St. Cruz, CA), respectively. Mouse anti-FLAG antibody, rabbit FITC-conjugated anti-mouse IgG secondary antibody and other chemicals unless noted otherwise were purchased from Sigma (St. Louis, MO).

### Cell lines and cultures

Human NIH:OVCAR-3 cell was purchased from the Bioresource Collection and Research Center (Hsinchu, Taiwan). TE1 and TE9 cells, originally purchased from the Japanese Collection of Research Bioresources, were kindly provided by Dr. Yann-Jang Chen (National Yang-Ming University, Taiwan). Ishikawa cell, originally purchased from the European Collection of Cell Culture, was kindly provided by Dr. Sin-Tak Chu (Academia Sinica, Taiwan). Other human cell lines, including HEK-293T, THP-1, KATO III, T-47D, RL95-2, HEC-1-A, HeLa and SKOV-3, were originally from ATCC.

### Plasmid construction and gene expression

The full-length sequences of *NMUR1*, *NMUR2* and *NMUR2S* were cloned from human placenta, brain and ovarian cDNAs (Clonetech, Palo Alto, CA), respectively. Human *LHR*-containing construct was kindly given by Dr. Aaron Hsueh (Stanford University, USA). The sequences encoding eGFP, DsRed and GST were amplified from pEGFP-N2, pDsRed-Monomer-N1 (Clonetech, Mountain View, CA) and pGEX-6P-1 (GE Healthcare Life Sciences, Piscataway, NJ) vectors, respectively. To construct the N-terminal tagged receptors, the first 16 amino acids of NMUR1 and the first 18 amino acids of NMUR2 or NMUR2S were substituted by a prolactin signal peptide (MNIKGSPWKGSLLLLLLVSNLLLCQSVAP) followed by an indicated epitope tag. To construct the C-terminal tagged receptors, the proteins were led by their original signal peptides and the designed tag was introduced using a PCR-based method. For receptor overexpression in HEK-293T cells, the cDNAs were cloned into the pcDNA3.1/Zeo (+) vector (Invitrogen) and the purified plasmids were transfected using the Turbofect transfection reagent (Thermo Scientific).

The stable cell lines expressing the FLAG-tagged NMU receptors were selected and maintained by zeocin. For overexpression of *eGFP* or *NMUR2S* in SKOV-3 cells, the cDNA encoding eGFP or human NMUR2S was subcloned into the pLAS2w.Ppuro vector. For knockdown of *GFP* and *NMUR2S* in THP-1 cells, the corresponding pLKO.1-puro lentivetors were purchased from Taiwan National RNAi Core Facility. The recombinant lentiviruses were obtained by cotransfection with pCMVΔR8.91, pMD.G (from the Taiwan National RNAi Core Facility) and lentivector construct into HEK-293T through Turbofect transfection reagent. The condition media were collected and used for further cell infection.

### Immunocytochemistry and fluorescence resonance energy transfer (FRET)

For immunocytochemical staining, the HEK-293T cells expressing FLAG-tagged NMU receptors were reseeded in the 12-well plates that contain a FBS-coated coverslip in each well for 48 hrs. After fixation with 4% paraformaldehyde in PBS for 30 mins at room temperature, the cells were incubated under permeable (0.5% Triton X-100 in PBS) or non-permeable (PBS only) conditions for 5 mins before further probed with the mouse anti-FLAG primary antibody and the rabbit anti-mouse IgG conjugated FITC secondary antibody. The cytoskeleton and nucleus of transfected cells were counter-stained with ALEXA FLUOR 568 phalloidin (Invitrogen) and DAPI (Invitrogen), respectively. The cell images were photographed with a confocal microscope (Olympus FluoView FV1000, Japan).

For the FRET assay, eGFP-tagged and DsRed-tagged receptors were co-expressed in HEK-293T cells. The transfected cells were grown on coverslip. The steps of fixation, permeabilization and mounting were performed as described above. An excitation wavelength of 488 nm and an emission range of 500 to 550 nm and an excitation wavelength of 543 nm and an emission range of 580 to 700 nm were used to acquire images of eGFP and DsRed, respectively. FRET was processed by using an acceptor photobleaching protocol against DsRed on a confocal microscope (Olympus FluoView FV1000, Japan). Briefly, DsRed was photobleached at an excitation wavelength of 543 nm for 3 mins in the whole cell. The fluorescence intensity of eGFP and DsRed was measured before and after the photobleaching of DsRed.

### Cell surface ELISA

The transfected HEK-293T cells or selected lines were grown in the 24-well plates followed by fixation with 4% paraformaldehyde in PBS for 30 mins at room temperature. After washed with 0.1% BSA in PBS, the cells were incubated with the mouse anti-FLAG primary antibody followed by the HRP-conjugated secondary antibody. The luminescence intensities were quantified after adding the chemiluminescent HRP substrate (Millipore, Billerica, MA).

### Western blotting and pull-down assay

For Western blotting against GPCRs, the cells were collected and incubated with the sample buffer designed for GPCR extraction (5% dithiothreitol, 4% sodium dodecyl sulfate, 8 M urea, 125 mM Tris-base, pH 6.8) at room temperature for 30 mins. The genomic DNAs were fragmented by sonication. The protein samples for ERK activation were collected by protein sample buffer (5% 2-mercaptoethanol, 2.5% sodium dodecyl sulfate, 10% glycerol, 125mM Tris-base, pH 6.8) and were boiled at 95°C for 10 mins.

For pull-down assay, the transfected HEK-293T cells were washed by cold PBS and lysed with the lysis buffer (300 mM sodium chloride, 50 mM Tris-base, 1.5 mM magnesium chloride, 1 mM calcium chloride, 10% glycerol, 1% Triton X-100, 0.1% sodium dodecyl sulfate, pH6.8) at 4°C for 1 hour. The supernatants of cell lysates were incubated with glutathione sepharose (Amresco Biosciences, Piscataway, NJ) at 4°C for 24 hrs. The sepharose was washed three times by lysis buffer and the receptor complex on the glutathione sepharose was eluted by the GPCR protein sample buffer. The above protein samples were analyzed by running 8% SDS-PAGE. Western blotting was performed using specific antibodies against FLAG, GST, phospho-ERK or total ERK.

### Radio-ligand binding assay

Human NMU peptide was iodinated using the iodination kit from Pierce Thermo Scientific. Receptor binding assays were conducted using 293T cells overexpressing NMU receptors (2x10^5^ viable cells per 500 μl). Cells were incubated in the binding buffer (PBS/0.1% BSA) with 300000 cpm ^125^I-NMU overnight at room temperature. For all reactions, nonspecific binding was measured by adding 1 μM unlabeled NMU for competition. After washing twice with the binding buffer, the amount of bound radioactivity in the cell pellet was determined by using a γ-counter.

### Reporter assay

For reporter assay, selected HEK-293T cells that stably express FLAG-tagged NMUR1 or NMUR2 were further co-transfected with pSRE-Luciferase (0.4 μg/ml) (Stratagene, La Jolla, CA), pCMV-β-Gal (0.2 μg/ml) and indicated NMU receptor plasmid (0.8 μg/ml) or control pcDNA3.1. One day after transfection, the media were replaced with serum-free DMEM supplemented with 0.1% BSA (5×10^5^ viable cells/ml) and treated with or without 100 nM human NMU for 16–18 hrs. After treatment, the cells were lysed with Glo lysis buffer (Promega Corp., Madison, WI) for 30 mins and the lysates were used for subsequent measurement of luciferase and β-galactosidase activity.

### cDNA preparation and gene quantification

For cDNA preparation, the total RNA from cancer cell lines or clinical materials were collected and extracted by TRIzol (Invitrogen) according to the manufacturer instructions. The cDNA of total mRNA were synthesized by High-Capacity cDNA Reverse Transcription Kits (Life technology, Grand island, NY) with oligo-dT primer. The primer pairs for the semi-quantitative PCR were listed as follows: human *NMU* forward, TCATTATTCGAAGACACAGAAGTTG; human *NMU* reverse, TACAACTGAGAACATTGACAACACA. Human *NMUR1* forward, CTGAGCGTGGAACGCTATGT; human *NMUR1* reverse, GATGGATCGGTCTCTTGCTG. Human *NMUR2* forward, CTCTACTACCTCATGGCACTCA; human *NMUR2* reverse, TCACTCGAGGGTTTTGTTAAAGTGGAAGC. Human *ACTB* forward, TGACAGACTACCTCATGAAGATCC; human *ACTB* reverse, CTGCT TGCTGATCCACATCTG. For the quantitative real-time PCR, Power SYBR Green PCR Master Mix was used and the primer pairs were listed as follows: human *NMU* forward, CTGCCGAGGTGCTCCAATA; human *NMU* reverse, CAATGGACAGAAAAGACGAACA. Human *NMUR1* forward, GCCGGAGACAAGTGACCAAGA; human *NMUR1* reverse, TGACACGACGCTCCACATG. Human *NMUR2* forward, CCTATTCTACCTCCTCCCCATGAC; human *NMUR2* reverse, CATTCCCTTCATCTGCCTCAA. Human *ACTB* forward, TCCTCCTGAGCGCAAG; human *ACTB* reverse, CTGCT TGCTGATCCACATCTG.

### Immunohistochemistry

The clinical materials of ovarian cancers and the paired adjacent normal tissues were fixed by Bouin’s solution following paraffin embedding. The antigens on 5 μm-thick sections were retrieved by Target Retrieval Solution, pH 9 (DAKO). For the performance of immunohistochmeistry, the rabbit polyclonal anti-NMU antiserum was prepared as previously described [18] and the rabbit polyclonal anti-NMUR2 antibody (Abcam) was used. The rabbit preimmune serum and the normal IgG (Cell Signaling) at the same concentration were used as negative controls, respectively. The staining of NMU and NMUR2 were performed using the Universal LSAB^TM+^ Kit/HRP (DAKO) and NovaRed HRP substrate kit (VECTOR) according to the manufacturers’ instructions.

### Proliferation assay

Human SKOV-3 and THP-1 cells were resuspended with FBS-containing media, counted and then seeded in 48-well plates (1500 cells/well for SKOV-3; 10000 cells/well for THP-1). To observe the cell viability of SKOV-3 at indicated intervals, 10% medium volume of AlamarBlue (AbD Serotec) was directly added in the wells and the plates were incubated for 3 h at 37°C. The media were transferred to black ELISA plate and the fluorescence intensities were measured with an excitation wavelength at 560 nm and emission wavelength at 590 nm. The cell numbers of THP-1 at indicated intervals were directly counted by hemocytometer. All experiments were performed in a triplicate manner.

### Data analysis

All experimental data are presented as means ± SD of triplicate cultures or samples. For all data, at least three individual repeated experiments were carried out, and these showed similar results. Statistical significance in individual group was determined by the Student’s *t*-test and data sets were examined by one-way ANOVA followed by a post-hoc Dunnett's t-test. For the gene expression in the clinical materials, the statistical significance was determined by the Mann-Whitney test. Significance was accepted at *P* < 0.05 and is indicated by asterisks.

## Results

### Isolation of the *NMUR2* splice variant, *NMUR2S*

**Fig 1:**
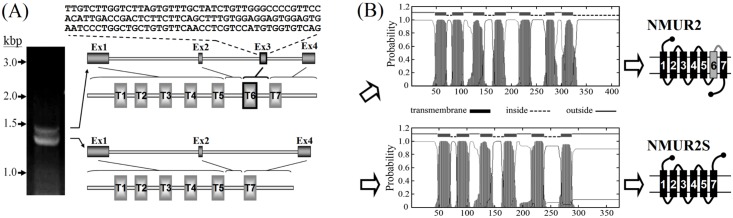
Cloning and structural prediction of NMUR2S. (A) Two splicing variants were obtained during amplification of the *NMUR2* gene from human ovarian cancer cDNA. As compared with the full-length *NMUR2*, the shorter variant, *NMUR2S*, lacks the third exon of *NMUR2*. (B) Sequencing data indicated that the encoding NMUR2S lacks the sixth transmembrane domain and the third extracellular loop of NMUR2; these are represented by the grey box and line in the right panel, respectively. Thus, NMUR2S is predicted to be a six-transmembrane protein with an extracellular C-terminus.

During PCR amplification of full-length *NMUR2* from a human ovarian cancer cDNA pool, a short but dominant splice variant was found and cloned (Fig 1A, left panel). Sequencing data indicated that alternative splicing of the mRNA had resulted in deletion of the third exon, which encodes 42 amino acids that originally compose the sixth transmembrane domain and the third extracellular loop of NMUR2 (Fig 1A, right panel). Unlike NMUR2, which consists of seven transmembrane domains and an intracellular C-terminus, the TMHMM 2.0 transmembrane prediction revealed that this novel truncated NMUR2, which we have called NMUR2S, has only six transmembrane domains and a long extracellular 85 amino acid C-terminal tail [23] (Fig 1B).

### Translation and configuration of the NMUR2S protein

**Fig 2:**
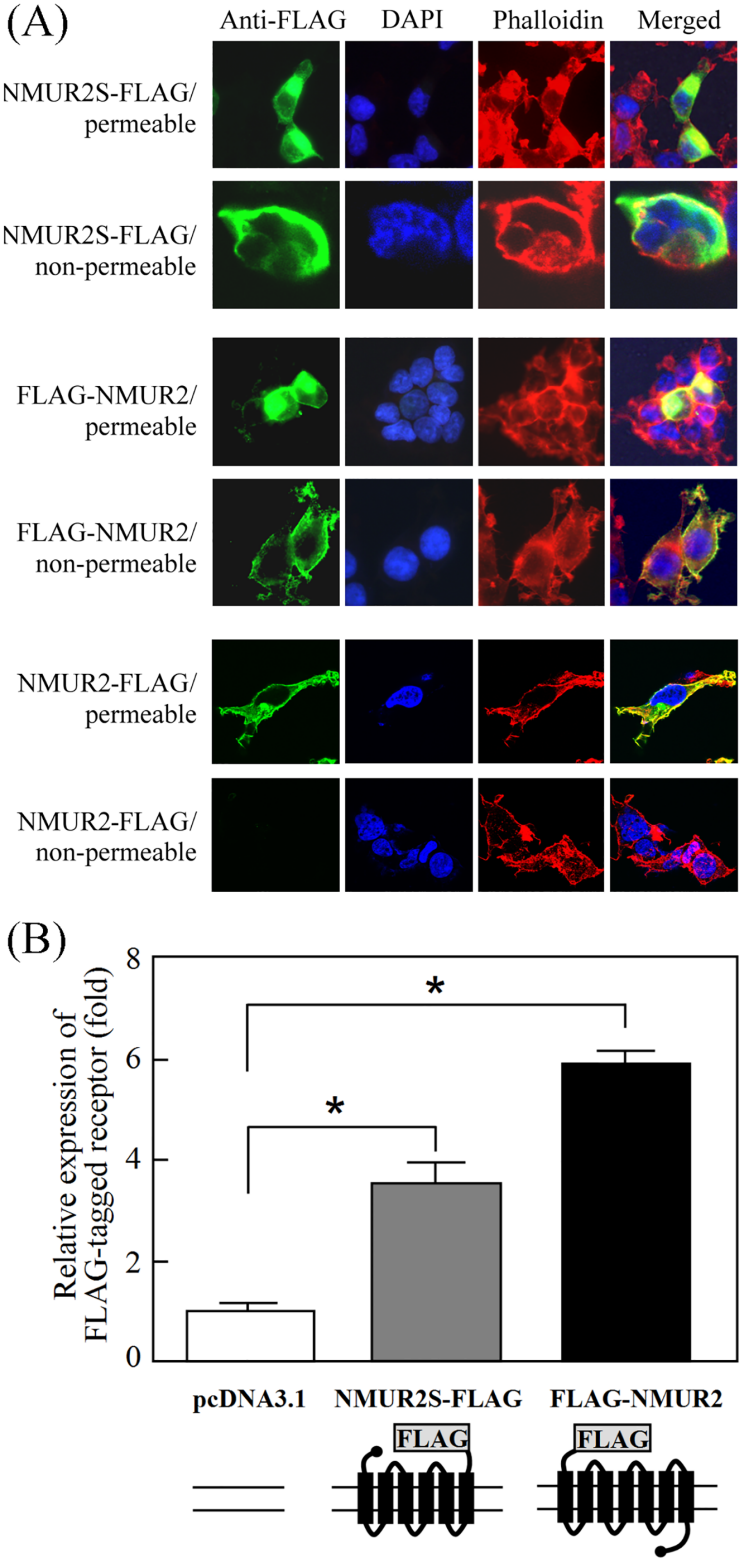
The membrane orientation of NMUR2S. (A) 293T cells expressing NMUR2S-FLAG, FLAG-NMUR2 or NMUR2-FLAG were probed with anti-FLAG antibody followed by FITC-conjugated secondary antibody under permeable (0.5% Triton X-100 in PBS) or non-permeable (PBS only) conditions. The distribution of receptor was observed by a confocal fluorescence microscope. DAPI and phalloidin were used for nuclear and cytoskeletal staining, respectively. (B) The cells were subjected to the cell-surface ELISA to compare the levels of the FLAG epitope on the cell surface. Data are shown as the mean ± SD. *, *P* < 0.05.

NMUR2S was predicted to be a novel six-transmembrane receptor with an extracellular C-terminus. To confirm this, immunocytochemical staining was performed using FLAG-tagged receptors. Like the extracellular N-terminal FLAG-tagged NMUR2, the C-terminal FLAG-tagged NMUR2S can be detected in both the plasma and ER membranes under permeable conditions but was only localized on the cell surface under non-permeable conditions (Fig 2A, upper and middle panels). In contrast, the C-terminal FLAG-tagged NMUR2 showed no staining under non-permeable conditions (Fig 2A, lower panel). Furthermore, the FLAG epitope tagged on the C-terminus of NMUR2S can also be detected and quantified using cell-surface ELISA (Fig 2B). Taken together, these results demonstrated that *NMUR2S*, although lacking the third exon that encodes the sixth transmembrane and the third extracellular loop of NMUR2, can still be translated, folded and translocated to the cell membrane successfully as a six-transmembrane receptor with its C-terminus outside the cell.

### Homodimerization and heterodimerization of the NMU receptors

**Fig 3:**
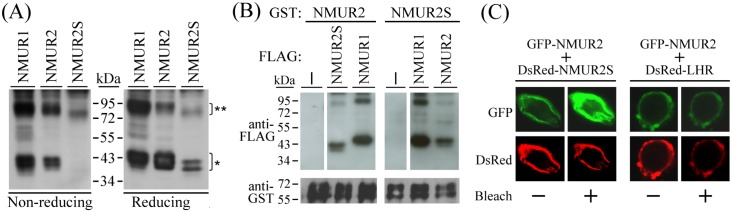
Dimerization relationship among NMU receptors. (A) 293T cells were transfected with FLAG-NMUR1, FLAG-NMUR2 or FLAG-NMUR2S individually for two days. Cells were harvested and then lyzed under non-reducing or reducing conditions for Western blotting against the FLAG epitope. The positions of receptor monomer (*) and homodimer (**) are indicated. (B) 293T cells were co-transfected with GST-tagged and FLAG-tagged receptors as indicated. The cell lysates were then subjected to the GST pull-down assay. The associated FLAG-tagged receptors and the GST pull-down efficiency were determined by Western blotting. The upper image was derived from the same blot but was processed to remove some unnecessary bands in between. (C) 293T cells were co-transfected with GFP-NMUR2 and either DsRed-NMUR2S or a control DsRed-LHR. During FRET measurement by confocal microscopy, the changes in GFP fluorescent intensity were captured and compared in each set before or after UV bleach of DsRed.

Formation of NMU receptor homodimers was first examined. We found that the forming NMU receptor complexes can only be extracted in mild conditions without boiling. In the extracts of 293T cells overexpressing N-terminal FLAG-tagged NMUR1, N-terminal FLAG-tagged NMUR2 or N-terminal FLAG-tagged NMUR2S, Western blotting against the FLAG epitope showed mainly two groups of immunoreactive signals under non-reducing conditions, with approximate molecular weights of around 80 kDa and 40 kDa (Fig 3A, left panel). Although the dimeric GPCR complexes involve multiple interactions and may not be separated completely in the GPCR extraction buffer without boiling, the band intensities still shifted apparently from 80 kDa to 40 kDa under reducing conditions (Fig 3A, right panel), suggesting these two positions correspond to the dimeric and monomeric forms of each NMU receptor, respectively. Sequence analysis in combination with structural prediction suggest that both NMUR2 and NMUR2S contain at least one N-linked glycosylation site, N^194^, locating extracellularly at the second extracellular loop, where it can be modified post-translationally; this may explain the double bands shown at their monomeric positions.

Next, we co-transfected two different tagged NMU receptors in 293T cells to evaluate their heterodimeric relationship. Pull-down assays demonstrated that NMUR2 can not only heterodimerize with NMUR2S but also with NMUR1. Likewise, NMUR2S and NMUR1 can also form a heterodimer (Fig 3B).

To exclude possible artifacts associated with nonspecific protein aggregation of membrane-bound receptors during detergent extraction in pull-down assays, an acceptor photobleaching FRET approach in intact cells, in which the emission of the donor will be increased after blocking the energy transfer by prebleaching the acceptor, was used. In 293T cells co-expressing GFP-NMUR2 and DsRed-NMUR2S, a significant increase in GFP emission was detected in cells after photobleaching the DsRed, indicating that these two receptors can interact with each other on the cell surface (Fig 3C, left panel). One concern that has always been raised in this field is that GPCR heterodimerization might be due to an artifact of receptor overexpression. To exclude this possibility, luteinizing hormone receptor, evolutionarily distant from NMU receptors, was used as a control. Contrary to the above result, no fluorescence recovery of the GFP donor was detected in the cells co-expressing GFP-NMUR2 and control DsRed-luteinizing hormone receptor after photobleaching the DsRed (Fig 3C, right panel); this supports the hypothesis that the interaction between the NMU receptors is specific. Taken together, the Western blotting, pull-down assays and FRET experiments all suggest that these three NMU receptors can not only form homodimers but also form heterodimers with each other.

### NMUR2S antagonizes NMU signaling

**Fig 4:**
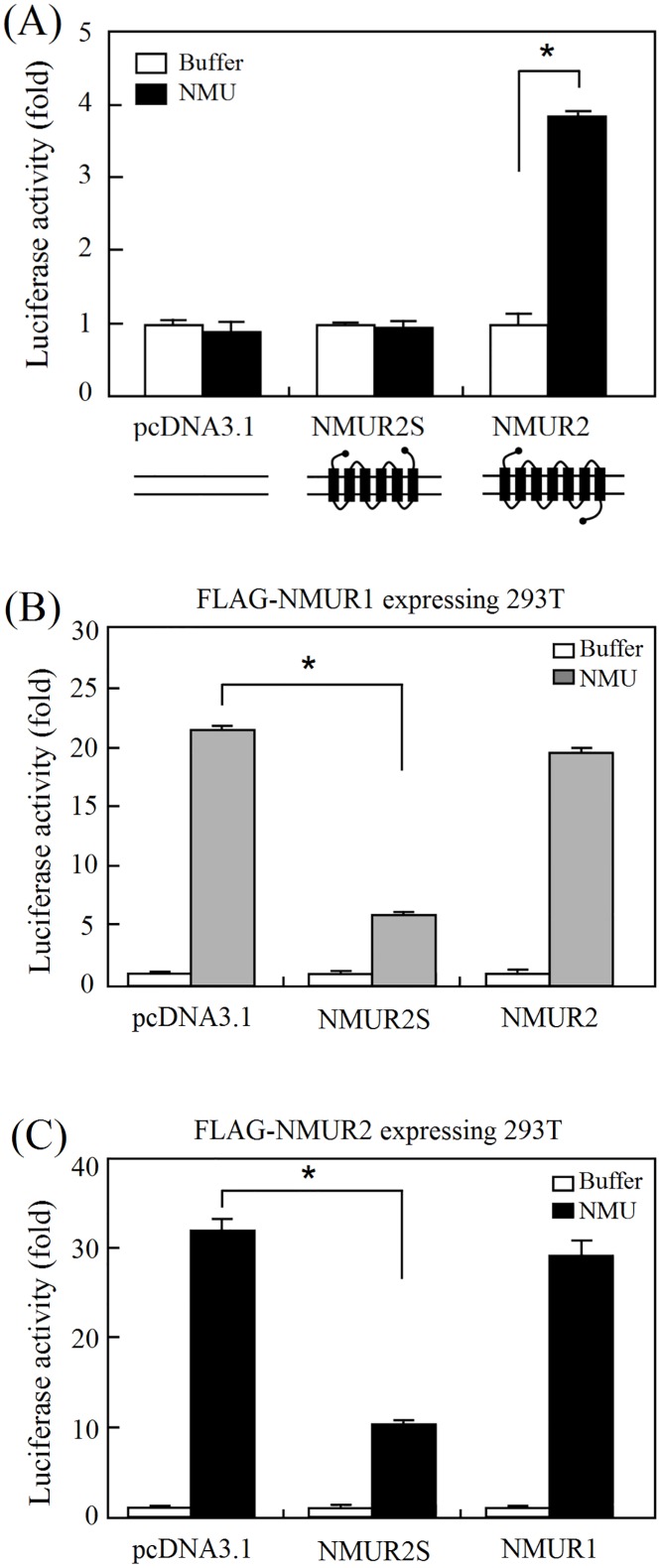
The inhibitory effect of NMUR2S on the transduction of NMU signaling. (A) 293T cells were transiently transfected with pSRE-Luc, pCMV β-Gal and pcDNA3.1, NMUR2S or NMUR2 overnight. The luciferase activity levels were determined and normalized against each β-galactosidase value after treatment with 100 nM NMU for 8 hr. To test the NMUR2S effects on the normal receptor signaling, 293T cells stably expressing (B) FLAG-NMUR1 or (C) FLAG-NMUR2 were further transfected with pSRE-Luc, pCMV β-Gal control and pcDNA3.1 or another NMU receptor construct as indicated. The cells were then treated with or without 100 nM NMU for 8 hr before measuring the luciferase activities. The value in cells without NMU stimulation was used as a one-fold control. Data are shown as the mean ± SD. *, *P* < 0.05.

NMUR2S is translocated well to the cell membrane (Fig 2) and is capable of heterodimerizing with NMUR2 and also NMUR1 (Fig 3). We therefore further characterized the roles of NMUR2S in conducting NMU signaling using a luciferase reporter assay [19]. In contrast to NMUR2, which can be activated by NMU in a dose-dependent manner, cells overexpressing NMUR2S showed no response to NMU (Fig 4A). Importantly, in 293T cells stably expressing N-terminal FLAG-tagged NMUR1, further transfection of NMUR2S, but not of NMUR2, showed an apparent decrease in the NMU response (Fig 4B). Likewise, NMUR2S, but not NMUR1, expression significantly dampened the NMU response in the NMUR2-expressing stable cell line (Fig 4C). Although not activated by NMU directly, these findings suggest that NMUR2S has a dominant negative effect on NMU signaling when it heterodimerizes with NMUR1 or NMUR2.

### The consequences of NMU receptor heterodimerization on receptor expression and ligand binding

**Fig 5:**
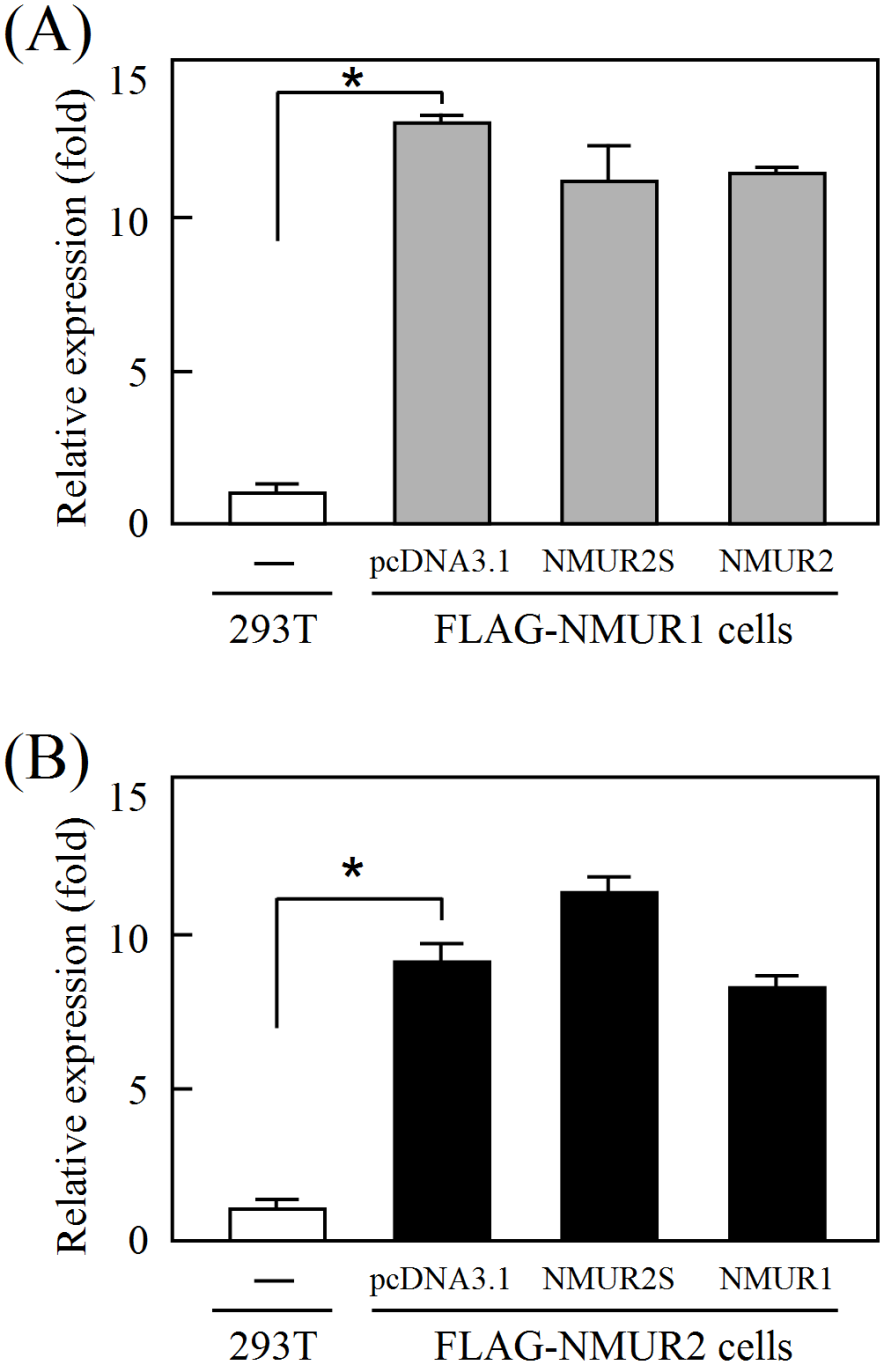
NMUR2S does not affect the membrane expression of NMUR1 and NMUR2. 293T cells stably expressing (A) FLAG-NMUR1 or (B) FLAG-NMUR2 were further transfected with pcDNA3.1 or other non-tagged NMU receptor plasmid as indicated. After overnight culture, the surface levels of FLAG-NMUR1 or FLAG-NMUR2 were determined by cell-surface ELISA using antibody against the FLAG epitope. The value in non-transfected 293T cells served as a one-fold background control. Data are shown as the mean±SD. *, *P* < 0.05.

Dimerization of NMUR2S with normal NMU receptors may potentially affect the surface expression level, ligand binding ability and/or internalization rate of functional receptors [11]. To clarify this, cell-surface ELISA was performed. In 293T cells stably expressing N-terminal FLAG-tagged NMUR1, further transfection with NMUR2S or with NMUR2 did not affect the cell-surface level of NMUR1 (Fig 5A). Similar results were also observed in cells stably expressing N-terminal FLAG-tagged NMUR2 (Fig 5B). In agreement with the cell-surface ELISA results that showed no change in the NMUR1 or NNMUR2 level, co-expression of NMUR2S does not seem to affect the translocation efficiency or internalization rate of normal NMU receptors.

**Fig 6:**
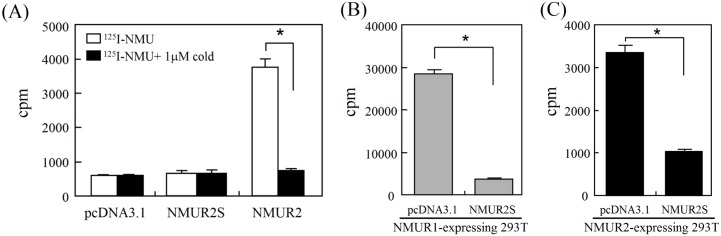
NMUR2S dampens the ligand binding of NMUR1 and NMUR2. (A) To test the ligand binding ability of NUR2S, 293T cells (10^6^ cells/tube) transfected with pcDNA3.1, NMUR2S or NMUR2 were incubated with iodinated NMU (100,000 cpm/tube) with or without 1 μM unlabeled NMU overnight. To test the effect of NMUR2S on ligand binding of NMUR1 and NMUR2, 293T cells stably expressing (B) NMUR1 or (C) NMUR2 were further transfected with pcDNA3.1 or NMUR2S. Cells were then harvested and incubated with iodinated NMU overnight. After washed, the amounts of bound radioactivity were determined using a γ-spectrometer. Data are presented as mean±SD. *, *P* < 0.05.

We further preformed radioligand-binding studies to determine whether NMUR2S alters the NMU binding capacity of normal NMU receptors. In contrast to NMUR2-transfected 293T cells, NMUR2S-transfected cells showed no detectable specific ligand binding using ^125^I-labeled NMU (Fig 6A). This may also explains why no downstream signaling was detected in NMUR2S-expressing cells when stimulated with NMU (Fig 4A). Importantly, in 293T cells stably expressing NMUR1 or NMUR2, the binding amount of ^125^I-labeled NMU decreased dramatically upon co-expression of NMUR2S (Fig 6B and 6C), which suggests that heterodimerization between NMUR2S and NMUR1 or between NMUR2S and NMUR2 is able to prevent the ligand binding to functional receptors and this then results in a significant dampening of NMU signaling.

### NMU signaling is up-regulated in ovarian cancers

**Fig 7:**
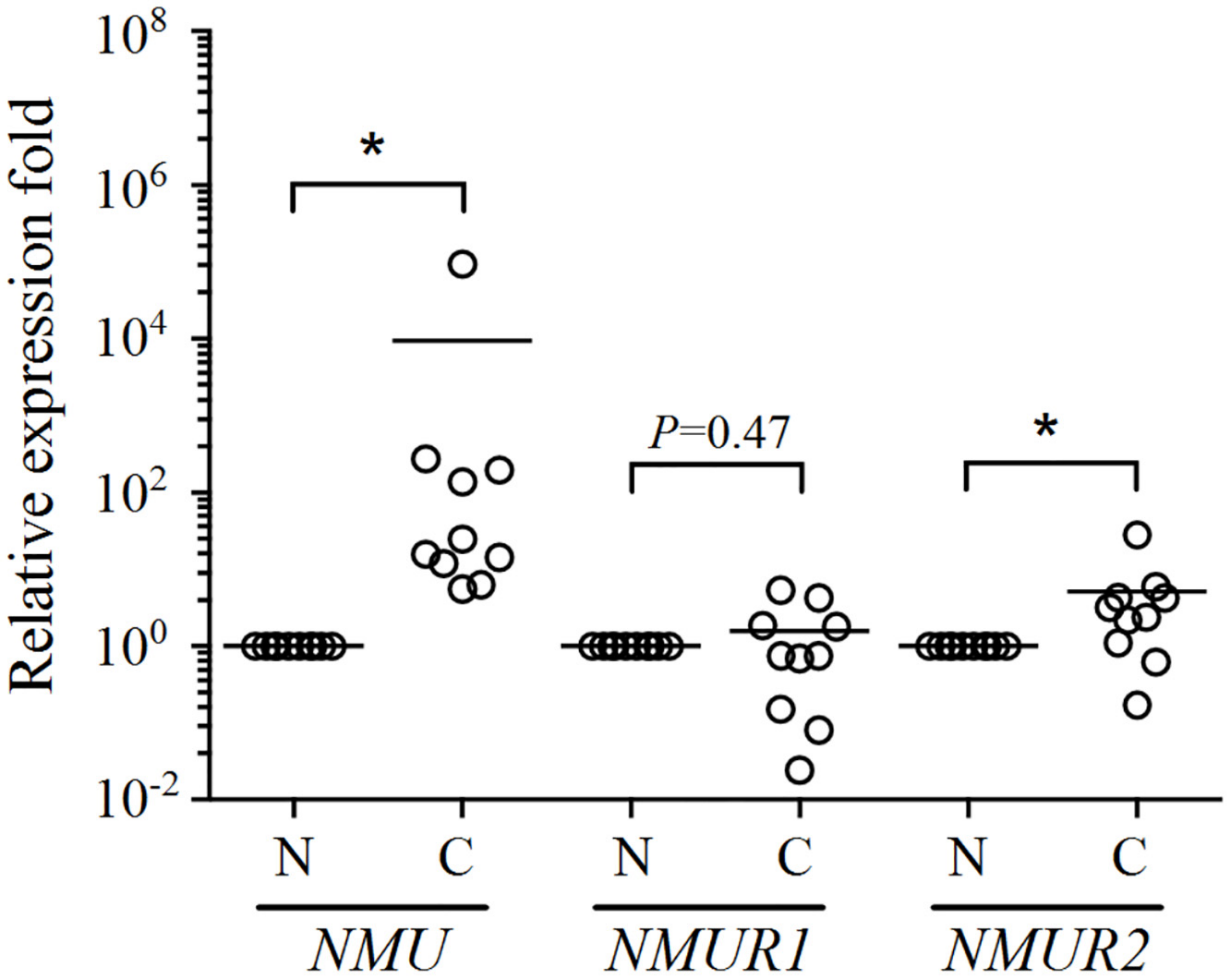
Expression of *NMU* and NMU receptors in human ovarian cancer tissues. *NMU*, *NMUR1* and *NMUR2* mRNA levels were quantified and compared between human ovarian cancer and adjacent normal tissues (n = 10). The relative gene levels in the cancer tissue samples were normalized against their paired adjacent normal for each patient and are shown as the log_10_ of the relative quantity. β-actin levels served as internal controls. *, *P* < 0.05.

**Fig 8:**
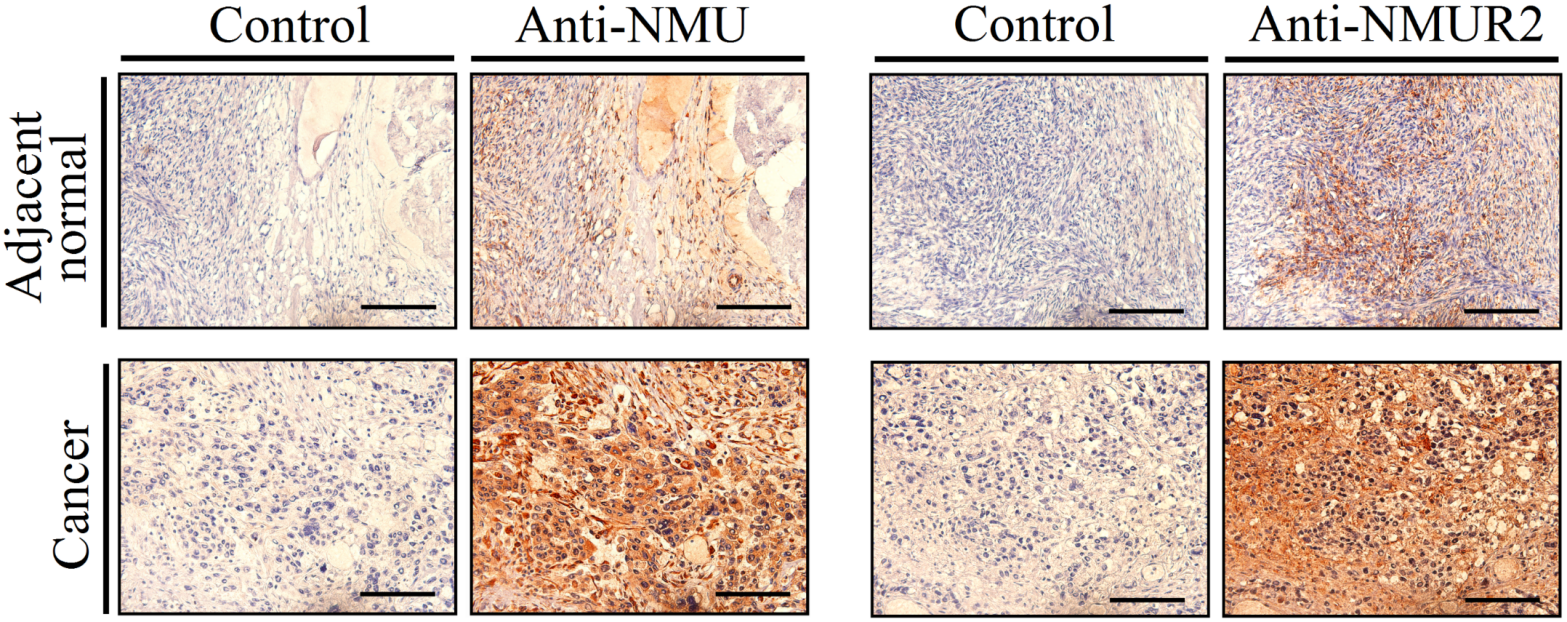
Immunohistochemical staining of NMU and NMUR2 in patient specimens. Represented sections from adjacent normal (upper panels) and cancer tissues (lower panels) of ovarian cancer patients were probed with control pre-immune serum, anti-NMU antibody, control IgG, or anti-NMUR2 antibody as indicated. The morphology was revealed by counter-staining with hematoxylin. Bars = 100 μm.

In addition to cell-based experiments, we also wanted to further explore whether the expression of NMUR2S can interfere in NMU signaling endogenously. We have previously demonstrated the existence of NMU signaling in the mammalian ovary [18]. Following this, we further found the transcripts of *NMU* and *NMUR2* are significantly induced in the ovarian cancer tissues; this increase was by 941 folds and 5 folds, respectively on average compared to those in the adjacent normal controls (Fig 7). To further evaluate any change in protein levels, specimens of a patient where the *NMU* and *NMUR2* transcript levels were close to the median of the test group were subjected to immunohistochemical staining. These results indicated that the NMU and NMUR2 immunoreactive signals in the ovarian cancer tissues are much stronger than those in the adjacent normal controls (Fig 8), consistent with their mRNA profiles as shown in Fig 7. NMU signaling has been proven to promote tumorigenesis in many cancers [24–27]. Taken together, up-regulation of both NMU and NMUR2 in ovarian cancers as shown by our findings suggests that over-activation of NMU signaling may be involved in promoting ovarian tumorigenesis. Therefore, it would be of interest to test the effect of NMUR2S on deterring the NMU-mediated progression of cancer cells, such as ovarian cancer cells.

### Change in the level of NMUR2S alters NMU signaling in cancer cells *in vitro*

**Fig 9:**
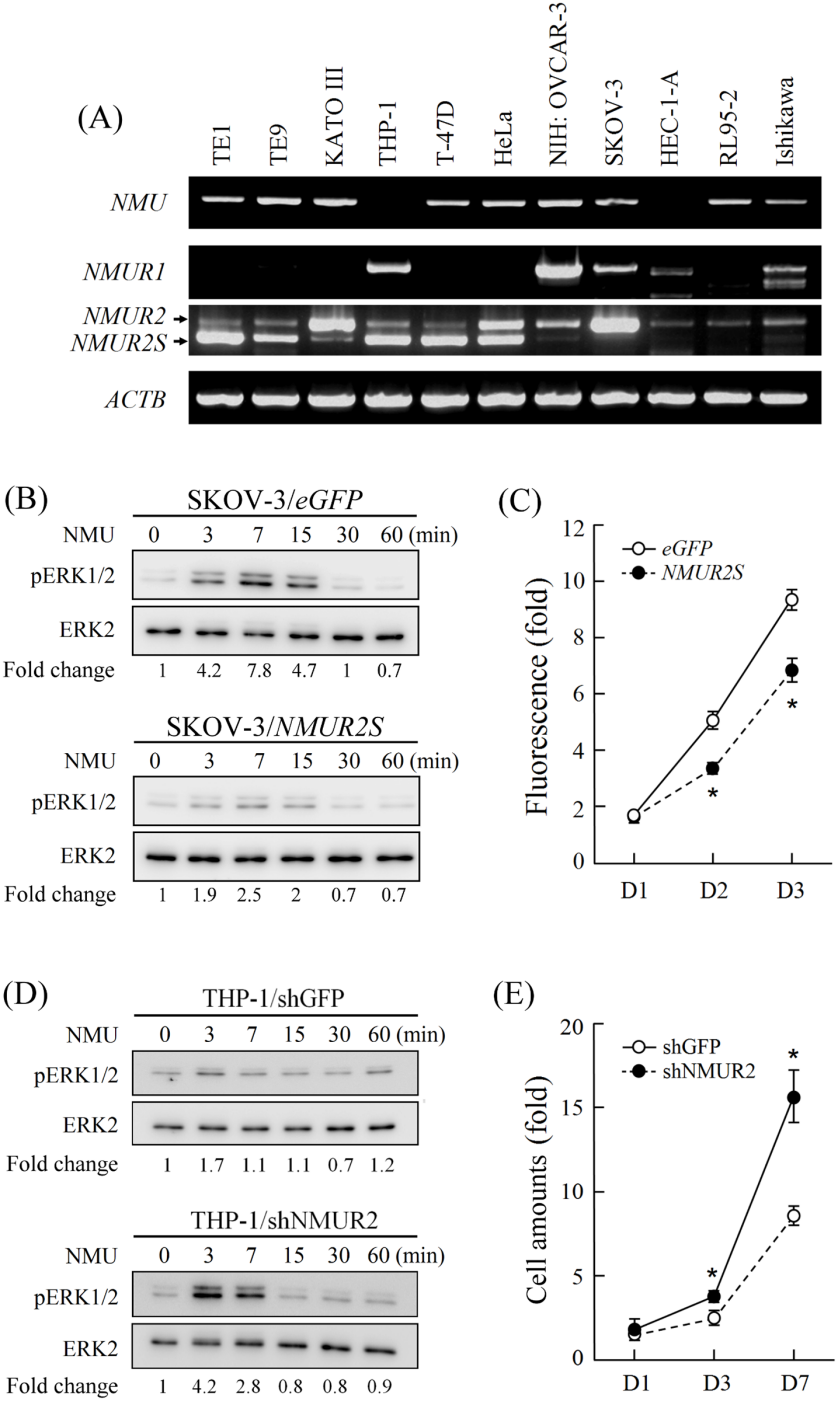
Expression of NMUR2S and its effects on cancer cell signaling and progression. (A) The transcripts of *NMU*, *NMUR1*, *NMUR2* and *NMUR2S* were compared in diverse cancer cell lines. β-actin (*ACTB)* levels served as loading controls. (B) SKOV-3 cells overexpressing *eGFP* or *NMUR2S*, or (D) THP-1 cells with *eGFP* or *NMUR2S* knockdown were then treated with 100 nM NMU for different intervals before estimating the amounts of phosphorylated ERK1/2 (pERK1/2) and total ERK 1/2 by Western blotting. The ratios of pERK1/2 to ERK 1/2 in each sample were then calculated by densitometry and are shown below. (C) Cell proliferation rates between *eGFP*-expressing and *NMUR2S*-expressing SKOV-3 cells were compared using the AlamarBlue assay. The fluorescence value of the cells on day 0 (D0) served as a one-fold control. (E) Cell proliferation rates between *GFP*-knockdown and *NMUR2S*-knockdown THP-1 cells were compared by counting the cell numbers directly. The amount of THP-1 cells on day 0 (D0) served as a one-fold control. *, *P* < 0.05.

To characterize the NMUR2S effect on cancer cell progression, the expressions of *NMU* and its two receptors were screened and they were found to show distinct profiles in various cancer cells (Fig 9A). Specifically, only monocytic leukemia-derived THP-1, ovarian carcinoma-derived NIH:OVCAR-3 and ovarian carcinoma-derived SKOV-3 exhibited a higher *NMUR1* expression level. Among them, only SKOV-3 also co-expressed *NMU* and *NMUR2* but showed a negligible amount of *NMUR2S*. Thus, only SKOV-3 is suitable as an ovarian cancer cell model for testing the effect of NMUR2S by overexpression strategies. In contrast to control cells that expressed *eGFP*, the induction efficiency of downstream ERK1/2 phosphorylation by NMU treatment was significantly decreased in cells overexpressing *NMUR2S* (Fig 9B), which suggests that there is a blockage of NMU signaling in these cells. In addition, *NMUR2S* overexpression can also decrease the proliferation rate of SKOV-3 cells (Fig 9C).

Conversely, THP-1, which expressed comparable levels of *NMUR1* and *NMUR2S*, is suitable to test the effect of NMUR2S by knockdown strategies. The results showed that depletion of *NMUR2S* by expressing shNMUR2 significantly restored the NMU effect on THP-1 cells as reflected by increase in downstream ERK1/2 phosphorylation and also cell proliferation rate (Fig 9D and 9E); this may be because of the recuperation of functional NMUR1. Taken together, these findings suggest that the presence of NMUR2S is able to block endogenous NMU receptor-mediated signaling in SKOV-3 and THP-1 cells, potentially through receptor heterodimerization, and this then can lead to a suppression of various tumorigenic effects, such as proliferation.

## Discussion

In this study, we identified a novel *NMUR2* splice variant that lacked the third exon; this leads to deletion of both the sixth transmembrane domain and the third extracellular loop and inversion of the seventh transmembrane domain of NMUR2. Thus, the derived NMUR2S is a six-transmembrane domain protein with an extracellular C-terminus (Figs 1 and 2). In accordance with the theory that GPCRs may heterodimerize with evolutionarily close members, we have also demonstrated that NMUR1, NMUR2 and this newly identified NMUR2S are able to form heteromeric complexes without the presence of NMU, which suggests that this process is constitutive and not modulated by ligand binding. Over recent years, many GPCR splice variants have been found. In many cases, the derived truncated receptors behave as dominant-negative molecules by preventing the expression of their respective normal receptors on the cell surface through receptor dimerization. For example, a six-transmembrane domain calcitonin receptor isoform, which is derived from a splice variant with a deletion of the thirteenth exon, exerts a dominant-negative effect on the calcitonin signaling by retaining the normal receptor in the ER through dimerization [28]. A similar effect has also been reported for the truncated mutants of dopamine D3 receptor [29], of gonadotropin-releasing hormone receptor [30], of V2 vasopressin receptor [31], of CCR5 chemokine receptor [32], of histamine H3 receptor [33] and of tachykinin NK2 receptor [34]. In contrast to the situation with these cases, we have demonstrated in this study that NMUR2S has a unique expression characteristic compared with other truncated GPCR isoforms; this is that it not only can express well on the plasma membrane but also does so without causing mislocalization or accelerating internalization of the normal NMU receptors (Figs 2 and 5).

Interestingly, the changes in the domain orientation of NMUR2S still allow the molecule to retain its intermolecular receptor interaction ability. This suggests the first five transmembrane domains of NMUR2 are already adequate for both receptor homodimerization and heterodimerization. Indeed, several studies have indicated that the transmembrane domains closest to the N terminal end of the protein are more important for receptor dimerization. If ghrelin receptor is taken as an example, a truncated ghrelin receptor that contains only the first to fifth transmembrane domains is able to restrict the conformational change of the full-length receptor and thus block the conduction of ghrelin signaling [35]. In addition, a truncated V2 vasopressin receptors containing at least the first three transmembrane domains can already act as negative regulators of wild-type receptor function [31]. In this study, we have also shown that NMUR2S not only can not bind NMU itself but also dampens the ligand binding capability of NMUR1 and NMUR2 in transfected 293T cells (Fig 6), suggesting that NMUR2S may interfere in the formation of ligand binding pocket of normal NMU receptors through receptor heterodimerization. Based on the conformational differences between NMUR2 and NMUR2S, these findings suggest that the third extracellular loop of NMUR2 may be critical for the formation of the ligand binding pocket. However, one can not exclude the involvement of the extracellular C-terminal tail of NMUR2S, which may provide steric or other yet uncharacterized effects on the blockage of ligand-receptor interaction.

Nevertheless, if the above assumption is true, expression of NMUR2S will directly decrease the efficacy of NMU receptors and may even change the ligand binding affinity of NMUR1 or NMUR2 in the heterodimeric complexes. It would be of interest to perform more experiments such as the dose-response signaling test and the ligand-binding assay in order to clarify these issues in details. Furthermore, although we have demonstrated that the translocation efficiency or internalization rate of NMUR1 and NMUR2 will not be affected by co-expression of NMUR2S in the cell-surface ELISA results (Fig 5), it can not be sure that these processes remain the same under the presence of ligand. Therefore, even though we concluded that the decrease in radio-ligand amounts shown in the cells co-expressing NMUR2S and NMUR1 or NMUR2 is mainly due to the structural interruption of ligand binding pocket of normal NMU receptors by NMUR2S, in the present binding assay that was performed overnight at room temperature, we can not totally excluded the possibilities of ligand internalization and subsequent degradation or processing, which may contribute partially to the radioactivity amounts counted in the cell pellets. More experiments by modifying the binding conditions, such as by shorting the incubation time or by decreasing the incubation temperature, may help to solve this puzzle.

We have previously demonstrated the existence of NMU signaling in the rat ovary, where NMU and NMUR2 compose a novel autocrine system that controls ovarian cell development as well as progesterone production [18]. Therefore, we further tried to clarify whether the splice variant *NMUR2S* also exists in rats or other mammals. Surprisingly, it can not be amplified from normal rat and mouse ovaries (data not shown), which suggests that the expression of *NMUR2S* may be species specific and/or can only be induced under certain pathological conditions or in specific tissues for the regulation of NMU signaling.

Based on our previous finding that NMU signaling is present in the ovary, here we further have shown that both the transcript and protein levels of NMU and NMUR2 were increased in ovarian cancer tissue samples (Figs 7 and 8). Overexpression of NMUR2S significantly suppresses NMU downstream signaling and the proliferation rate of SKOV-3 ovarian cancer cell line, which expresses *NMUR2* dominantly and *NMUR1* moderately (Fig 9A). Taken together, these findings suggest that up-regulation of NMU signaling may help promoting ovarian cancer progression and that this effect can be reversed by introduction of NMUR2S, which can potentially heterodimerize with NMUR2 and NMUR1 to dampen NMU signaling. Thus, expression of *NMUR2S* may have pathophysiological consequences to regulate NMU signaling. Indeed, we did demonstrate that *NMUR2S* is co-expressed together with *NMUR2* in both the cancer and adjacent normal tissues harvested from patients with ovarian cancers. In addition, *NMUR2S* also co-exists with *NMUR2* and/or *NMUR1* in many other human cancer cell lines (Fig 9A). Whether it modulates NMU signaling in these cancers and cancer cell lines needs more studies.

Interestingly and similar to our findings in the ovarian cancer, earlier studies have also demonstrated that NMU signaling is up-regulated in diverse cancers and seems to play a role in promoting tumorigenesis. For example, the *NMU* gene is specifically up-regulated in non-small cell lung cancers, whereas depletion of *NMU* expression inhibits the growth of the lung cancer cells. [27]. In addition, NMU signaling has also been suggested to promote tumorigenesis in leukemia, bladder cancer and pancreatic cancer [21, 24–26]. Based on our findings, expression of NMUR2S can not only block NMUR2 activation but also NMUR1 activation through receptor heterodimerization. Therefore, induction of the alternative splicing mechanism to produce *NMUR2S* endogenously in these cancers may become a novel therapeutic strategy in the future.

Although approximate 50% of GPCR genes have been estimated to have alternative splicing forms [36, 37], the mechanisms that have been suggested for controlling the splicing machinery for GPCRs are very limited. We identified *NMUR2S* from the human ovarian cancer cDNA and have also previously showed that the *NMUR2* level can be regulated by gonadotropin signaling in the ovary [18, 38], the prominent organ for steroid hormone production. It is of interest that several reports have discovered that steroid levels are involved in modulating the alternative splicing of some GPCRs. Karteris *et al*. have observed that expression of an alternative splice variant of corticotropin-releasing hormone type 1β receptor can be increased by estradiol, but is decreased by progesterone [39]. In the rat brain and in pituitary-derived adenoma cells, the ratio of splice variant to normal dopamine D2 receptor has also been reported to be altered by circulating sex steroid levels [40]. It has been widely accepted that dysregulation of steroid signaling is highly related to cancer development in many organs including the ovary. Therefore, it will be interesting to further explore whether changes in steroid levels, or other as yet uncharacterized factors, during normal ovarian development or during ovarian tumorigenesis may play a role in modulating the alternative splicing machinery of *NMUR2*. Taken as a whole, detecting the level of *NMUR2S* and understanding the post-transcriptional mechanisms that control *NMUR2S* production during cancer progression could be of pivotal importance for future *in vivo* targeting of NMU signaling-related cancers both diagnostically and therapeutically.
